# 11β-Hydroxysteroid dehydrogenase type 1 inhibition in idiopathic intracranial hypertension: a double-blind randomized controlled trial

**DOI:** 10.1093/braincomms/fcz050

**Published:** 2020-01-10

**Authors:** Keira Markey, James Mitchell, Hannah Botfield, Ryan S Ottridge, Tim Matthews, Anita Krishnan, Rebecca Woolley, Connar Westgate, Andreas Yiangou, Zerin Alimajstorovic, Pushkar Shah, Caroline Rick, Natalie Ives, Angela E Taylor, Lorna C Gilligan, Carl Jenkinson, Wiebke Arlt, William Scotton, Rebecca J Fairclough, Rishi Singhal, Paul M Stewart, Jeremy W Tomlinson, Gareth G Lavery, Susan P Mollan, Alexandra J Sinclair

**Affiliations:** 1 Institute of Metabolism and Systems Research, College of Medical and Dental Sciences, University of Birmingham, Birmingham B15 2TT, UK; 2 Centre for Endocrinology, Diabetes and Metabolism, Birmingham Health Partners, Birmingham B15 2TH, UK; 3 Department of Neurology, University Hospitals Birmingham NHS Foundation Trust, Queen Elizabeth Hospital, Birmingham B15 2WB, UK; 4 Institute of Inflammation and Ageing, College of Medical and Dental Sciences, University of Birmingham, Birmingham B15 2TT, UK; 5 Birmingham Clinical Trials Unit, Institute of Applied Health Research, College of Medical and Dental Sciences, University of Birmingham, Birmingham B15 2TT, UK; 6 Birmingham Neuro-Ophthalmology, University Hospitals Birmingham NHS Foundation Trust, Queen Elizabeth Hospital, Birmingham B15 2WB, UK; 7 Department of Neurology, The Walton Centre NHS Foundation Trust, Liverpool L9 7LJ, UK; 8 Institute of Neurological Sciences, Queen Elizabeth University Hospital, NHS Greater Glasgow and Clyde, Glasgow G51 4TF, UK; 9 Nottingham Clinical Trials Unit, Queens Medical Centre, Nottingham NG7 2UH, UK; 10 Emerging Innovations Unit, Discovery Sciences, BioPharmaceuticals R&D, AstraZeneca, Cambridge CB2 0SL, UK; 11 Upper GI Unit and Minimally Invasive Unit, Heartlands Hospital, University Hospitals Birmingham NHS Foundation Trust, Birmingham B9 5SS, UK; 12 Medical School, University of Leeds, Leeds LS2 9JT, UK; 13 Oxford Centre for Diabetes, Endocrinology & Metabolism (OCDEM), NIHR Oxford Biomedical Research Centre, University of Oxford, Churchill Hospital, Headington, Oxford OX3 7LJ, UK

**Keywords:** idiopathic intracranial hypertension, 11β-hydroxysteroid dehydrogenase type 1, papilloedema, phase II, randomized controlled trial

## Abstract

Treatment options for idiopathic intracranial hypertension are limited. The enzyme 11β-hydroxysteroid dehydrogenase type 1 has been implicated in regulating cerebrospinal fluid secretion, and its activity is associated with alterations in intracranial pressure in idiopathic intracranial hypertension. We assessed therapeutic efficacy, safety and tolerability and investigated indicators of *in vivo* efficacy of the 11β-hydroxysteroid dehydrogenase type 1 inhibitor AZD4017 compared with placebo in idiopathic intracranial hypertension. A multicenter, UK, 16-week phase II randomized, double-blind, placebo-controlled trial of 12-week treatment with AZD4017 or placebo was conducted. Women aged 18–55 years with active idiopathic intracranial hypertension (>25 cmH_2_O lumbar puncture opening pressure and active papilledema) were included. Participants received 400 mg of oral AZD4017 twice daily compared with matching placebo over 12 weeks. The outcome measures were initial efficacy, safety and tolerability. The primary clinical outcome was lumbar puncture opening pressure at 12 weeks analysed by intention-to-treat. Secondary clinical outcomes were symptoms, visual function, papilledema, headache and anthropometric measures. *In vivo* efficacy was evaluated in the central nervous system and systemically. A total of 31 subjects [mean age 31.2 (SD = 6.9) years and body mass index 39.2 (SD = 12.6) kg/m^2^] were randomized to AZD4017 (*n* = 17) or placebo (*n* = 14). At 12 weeks, lumbar puncture pressure was lower in the AZD4017 group (29.7 cmH_2_O) compared with placebo (31.3 cmH_2_O), but the difference between groups was not statistically significant (mean difference: −2.8, 95% confidence interval: −7.1 to 1.5; *P *=* *0.2). An exploratory analysis assessing mean change in lumbar puncture pressure within each group found a significant decrease in the AZD4017 group [mean change: −4.3 cmH_2_O (SD = 5.7); *P *=* *0.009] but not in the placebo group [mean change: −0.3 cmH_2_O (SD = 5.9); *P *=* *0.8]. AZD4017 was safe, with no withdrawals related to adverse effects. Nine transient drug-related adverse events were reported. One serious adverse event occurred in the placebo group (deterioration requiring shunt surgery). *In vivo* biomarkers of 11β-hydroxysteroid dehydrogenase type 1 activity (urinary glucocorticoid metabolites, hepatic prednisolone generation, serum and cerebrospinal fluid cortisol:cortisone ratios) demonstrated significant enzyme inhibition with the reduction in serum cortisol:cortisone ratio correlating significantly with reduction in lumbar puncture pressure (*P* = 0.005, *R* = 0.70). This is the first phase II randomized controlled trial in idiopathic intracranial hypertension evaluating a novel therapeutic target. AZD4017 was safe and well tolerated and inhibited 11β-hydroxysteroid dehydrogenase type 1 activity *in vivo*. Reduction in serum cortisol:cortisone correlated with decreased intracranial pressure. Possible clinical benefits were noted in this small cohort. A longer, larger study would now be of interest.

## Teaser

Preclinical research highlighted the role of 11β-hydroxysteroid dehydrogenase type 1 inhibition in modulating intracranial pressure. We report the first phase II randomized clinical trial in idiopathic intracranial hypertension evaluating safety, tolerability, *in vivo* efficacy and initial clinical efficacy of a novel inhibitor of 11β-hydroxysteroid dehydrogenase type 1 to lower intracranial pressure in active idiopathic intracranial hypertension.

## Graphical Abstract

**Graphical Abstract:**
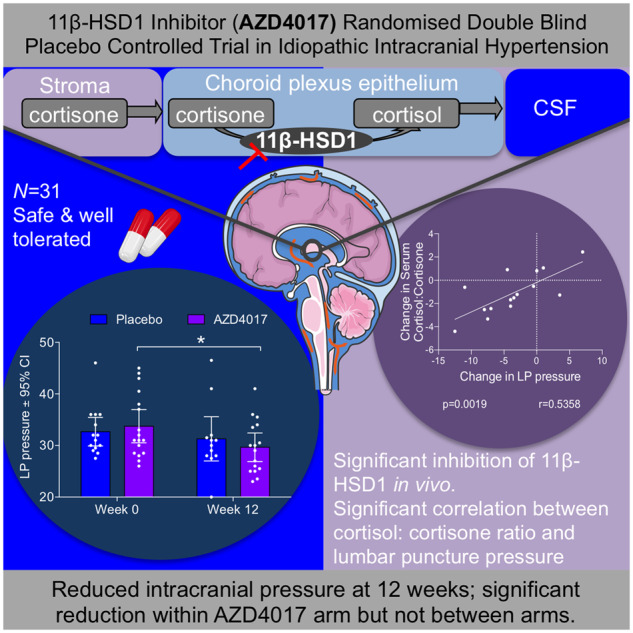

## Introduction

Idiopathic intracranial hypertension (IIH) is a debilitating condition characterized by raised intracranial pressure (ICP), papilledema, with the risk of permanent visual loss (Mollan *et al.*, 2018*b*) and chronic headaches, which reduce the quality of life (Mulla *et al.*, 2015). IIH predominately affects obese women between the ages of 25 and 36 years with a distinct androgen excess signature recently identified (Daniels *et al.*, 2007; Markey *et al.*, 2016; O'Reilly *et al.*, 2019). Incidence is increasing in line with escalating worldwide obesity rates (Mollan *et al.*, 2018*a*).

Surgical treatment is recommended when vision rapidly declines (Mollan *et al.*, 2014, 2018*c*), but the majority of patients (93%) are managed conservatively (Hoffmann *et al.*, 2018; Mollan *et al.*, 2018*a*, *b*). Dietary interventions are an effective treatment (Sinclair *et al.*, 2010*a*); however, meaningful and sustained weight loss is difficult to achieve (Colquitt *et al.*, 2014; Manfield *et al.*, 2017). Pharmacotherapy in IIH is limited (Piper *et al.*, 2015), with only two previous randomized controlled trials (RCTs) in IIH previously reported, both evaluating acetazolamide (Ball *et al.*, 2011; NORDIC Idiopathic Intracranial Hypertension Study Group Writing Committee *et al.*, 2014). New treatment options are therefore urgently required (Mollan *et al.*, 2018*b*).

We have previously demonstrated that the enzyme 11β-hydroxysteroid dehydrogenase type 1 (11β-HSD1) is expressed and active in the choroid plexus to amplify cortisol availability and acts to regulate cerebrospinal fluid (CSF) production (Sinclair *et al.*, 2007, 2010*b*; Gathercole *et al.*, 2013). In patients with IIH, resolution of disease (reduced ICP, improvements in papilledema and headaches) was associated with reduced 11β-HSD1 activity (Sinclair *et al.*, 2010*a*, *b*), with a study suggesting that inhibition of 11β-HSD1 with a non-selective inhibitor lowered intraocular pressure. Importantly, 11β-HSD1 expression and activity are dysregulated in obesity (Wake and Walker, 2004; Sandeep *et al.*, 2005).

Selective inhibitors of 11β-HSD1 have been developed as treatments for obesity, hepatic steatosis, metabolic syndrome and type 2 diabetes (Boyle, 2008; Stefan *et al.*, 2014). Based on these data, 11β-HSD1 could represent a therapeutic target for lowering CSF pressure. AZD4017 is a highly selective, fully reversible, competitive 11β-HSD1 inhibitor. It has been tested over short time intervals in healthy males (9 days) and abdominally obese subjects (10 days) and found to be safe and tolerable (AstraZeneca, 2000*a*, *b*, *c*, *d*, *e*). The ability of AZD4017 to penetrate the blood–brain barrier is not established; however, the choroid plexus lies outside the blood–brain barrier and consequently can be targeted directly following oral administration (Davson, 1966; Eftekhari *et al.*, 2015).

We hypothesized that the inhibition of 11β-HSD1 could be therapeutically beneficial in IIH. To test this theory, we conducted a multicenter, phase II double-blind, placebo-controlled RCT in IIH using the selective 11β-HSD1 inhibitor AZD4017, aiming to assess therapeutic efficacy, safety and tolerability, and investigated *in vivo* systemic and central nervous system efficacy.

## Materials and methods

### Study conduct

**Figure 1:**
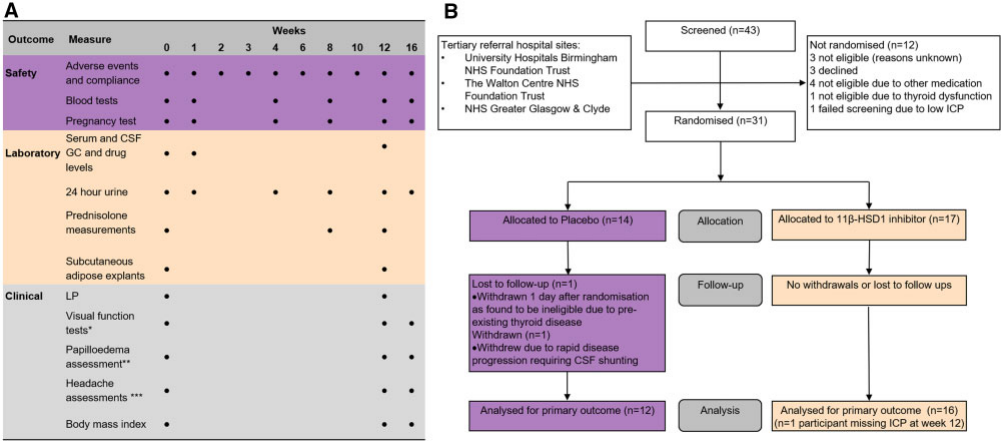
**Participant visits (**A**) and CONSORT diagram (**B**).** GC = glucocorticoids.

The study was conducted from March 2014 to December 2016 in three UK hospitals (Fig. 1). The National Research Ethics Committee York and Humber-Leeds West gave ethical approval (13/YH/0366). *In vitro* subcutaneous and omental adipose explants were collected from a separate IIH population undergoing bariatric surgery (National Research Ethics Committee Black Country 14/WM/0011). All patients provided written informed consent in accordance with the declaration of Helsinki. Detailed clinical trial methodology has been published (Markey *et al.*, 2017).

### Study population

Women (18–55 years) were eligible if they had a clinical diagnosis of active IIH meeting the updated, modified Dandy criteria (ICP > 25 cmH_2_O and active papilledema) and normal brain imaging (including magnetic resonance venography or CT with venography) at recruitment (for detailed eligibility criteria see Supplementary Table 1; Friedman *et al.*, 2013; Markey *et al.*, 2017).

### Study design

This study was a 16-week phase II, UK, multicenter, double-blind, placebo-controlled RCT with a 12-week dosing duration and 4-week follow-up of drug.

### Randomization and blinding

Participants were allocated to either the study drug or placebo using a trial number randomly allocated by phone, using block-of-6 randomization. Randomization was performed by an independent manufacturer (Almac) (Markey *et al.*, 2017). Participants and investigators were masked to treatment allocation during the trial.

### Intervention

Participants received 400 mg of an oral selective 11β-HSD1 inhibitor, AZD4017, twice daily for 12 weeks, compared with a matched placebo. Trial dosing was added to existing therapy for IIH; other drugs were maintained at a fixed dose throughout the study. Patients with IIH are typically informed about the importance of weight loss to treat their IIH as part of routine standard of care at diagnosis; however, they were not treated with an additional weight management intervention during this trial.

### Assessments

Participants completed the follow-up assessments at 1, 2, 3, 4, 6, 8, 10, 12 and 16 weeks (Fig. 1A).

### Clinical assessments

The primary outcome for clinical efficacy was the difference in ICP between AZD4017 and placebo, as measured by lumbar puncture at 12 weeks. Secondary outcomes included IIH symptoms, visual function [visual acuity measured using log of the minimum angle of resolution, perimetric mean deviation (PMD) using Humphrey 24-2 central threshold automated perimetry and contrast sensitivity assessed by MARs charts; Mars Perceptrix, USA], papilledema, headache-associated disability and anthropometric measures. Papilledema was evaluated using spectral domain optical coherence tomography (Spectralis, Heidelberg Engineering) to quantify the peripapillary retinal nerve fibre layer (RNFL) average and maximal values. Papilledema was graded from fundal photographs by three masked neuro-ophthalmologists using the Frisen classification (0 denotes no papilledema to grade 5 severest papilledema) (Frisen, 1982). Headache was evaluated through the headache impact test-6 disability questionnaire, headache severity (verbal rating scores 0–10), frequency (days per month), duration and analgesic use (days per month) (Bayliss *et al.*, 2003). Pill counting at each visit determined drug compliance.

In the original grant application and early versions of the protocol, the primary outcome measure was stated as the change in ICP between baseline and 12 weeks. Following adoption of the study by the Birmingham Clinical Trials Unit, the primary outcome was changed to ICP at 12 weeks, with adjustment for baseline ICP in the analysis. This change was made blind to any data analysis.

### Safety and tolerability

Adverse events and safety bloods were monitored (timeline Fig. 1A) including renal function (urea, creatinine and electrolytes), liver function (aspartate transaminase, alanine transferase, bilirubin, alkaline phosphatase, gamma-glutamyl transferase), thyroid function (thyroid-stimulating hormone, free thyroxine) and creatine kinase. Hypothalamic pituitary adrenal axis activity was monitored (cortisol, adrenocorticotropic hormone, dehydroepiandrosterone sulphate, testosterone, androstenedione, follicle-stimulating hormone, luteinizing hormone, oestradiol and progesterone).

Stopping criteria for the trial would occur if more than one participant met Hy’s law criteria or 10% or more had bilirubin levels over two times the upper limit of normal or alanine transaminase and/or aspartate transaminase over five times the upper limit of normal for 7 days, and no other cause for the liver dysfunction could be identified (Markey *et al.*, 2017).

### Glucocorticoid and AZD4017 blood and cerebrospinal fluid levels

Samples were collected and stored at −80°C. Cortisol and cortisone levels in serum and CSF were measured by liquid chromatography–tandem mass spectrometry (LC–MS/MS) at the University of Birmingham, as previously described (Hassan-Smith *et al.*, 2015; Long *et al.*, 2016). Plasma and CSF AZD4017 levels were quantified by an external laboratory (Alderley Analytical, Knutsford, UK).

### *In v* *ivo* systemic 11β-hydroxysteroid dehydrogenase activity

Global 11β-HSD1 activity was evaluated through the quantification of 24-hour urinary glucocorticoid metabolites, by LC–MS/MS (Sagmeister *et al.*, 2018). 11β-HSD1 activity was inferred from the ratio of (5α-tetrahydrocortisol + tetrahydrocortisol):tetrahydrocortisone [(5α-tetrahydrocortisol + tetrahydrocortisol):tetrahydrocortisone] alongside a stable ratio of total urinary cortisol (F):total urinary cortisone (E) reflecting 11β-HSD2 activity (Tomlinson and Stewart, 2001).

### *In vivo* hepatic 11β-hydroxysteroid dehydrogenase activity

Inhibition of hepatic 11β-HSD1 activity was informed by measuring first-pass metabolism of 10 mg of oral prednisone to prednisolone. Serum prednisone and prednisolone were measured every 20 minutes over 4 hours using LC–MS/MS (Richards *et al.*, 2012; Hassan-Smith *et al.*, 2015).

### *Ex vivo* adipose 11β-hydroxysteroid dehydrogenase activity

Subcutaneous adipose biopsies (*n* = 11 paired samples from baseline and 12 weeks, weight 100–150 mg in triplicate) were incubated in media (Dulbecco’s Modified Eagle’s Medium/Nutrient Mixture F-12; ThermoFisher, Rugby, UK) at room temperature with 100 nM cortisone (Sigma-Aldrich, Dorset, UK), with three media controls (without adipose) for 24 hours. Steroid conversion was quantified using LC–MS/MS (Juhlen *et al.*, 2015; Mooij *et al.*, 2015).

### *In vitro* adipose 11β-hydroxysteroid dehydrogenase inhibition by AZD4017

Subcutaneous and omental adipose explants (1–2 g in triplicate) were obtained from patients with IIH undergoing bariatric surgery. Samples were incubated with 2000, 200 or 20 nM of AZD4017 and 100 nM of cortisone alongside three controls (without AZD4017) for 24 hours. Steroid conversion was quantified using LC–MS/MS (Juhlen *et al.*, 2015; Mooij *et al.*, 2015).

### Statistical analysis

Analysis of the clinical data was based on the full analysis set according to the statistical analysis plan (Supplementary material). Analysis was conducted using intention-to-treat with data from all available randomized participants used. The primary comparison was between AZD4017 versus placebo at 12 weeks. The majority of data was continuous, so groups were compared using linear regression models with baseline measurements included as a covariate in the model. IIH symptom data were binary and were analysed using log-binomial models with baseline symptom included as a covariate in the model. The primary analysis of visual data included data from both eyes, using a linear mixed model with participant included as a random effect. We also analysed data from the most affected eye at baseline as defined by PMD (Friedman *et al.*, 2014). Statistical significance was set at *P* < 0.05, with no adjustment for multiple comparisons made. Clinical data were analysed using SAS (version 9.4) and STATA (version 14).

Analysis of laboratory data was performed using SPSS (version 24; IBM, New York, NY, USA). All laboratory data were continuous. The primary comparison between groups used an unpaired *t*-test for normally distributed data (Mann–Whitney *U* test for non-parametric data). For within-group comparisons (e.g. comparing baseline with 12-week data in one group), either the paired *t*-test or Wilcoxon signed-rank test was used for parametric or non-parametric data, respectively. We reported mean and standard deviation for parametric data (medians and ranges for non-parametric data).

### Sample size

To detect a difference between groups of 14% in ICP (assuming a standard deviation of 10% for ICP) with 90% power and two-sided alpha = 0.05, required 12 participants per group. Allowing for 20% drop out, we aimed to recruit 30 participants.

### Data availability

The trial is registered at Clinicaltrials.gov NCT02017444; European Clinical Trials Database (EudraCT Number: 2013-003643-31). The data that support the findings of this study are available from the corresponding author, upon reasonable request.

## Results

A total of 31 participants were recruited: 17 participants were randomized to AZD4017 and 14 participants were randomized to placebo (Fig. 1B). Baseline characteristics represent the cohort of patients with IIH with active disease recruited (Table 1). Baseline characteristics were not significantly different between trials arms, although mean deviation differed between groups (AZD4017: −3.4 db versus placebo: 6.1 db; *P* = 0.077). Acetazolamide was continued at a stable dose in 32% of participants (balanced between the trial arms; Table 1) and no other pharmacological IIH treatments were taken by the trial cohort.

**Table 1:** Baseline characteristics and ophthalmic measurements

	Placebo (*n* = 14)	AZD4017 (*n* = 17)	Total (*n* = 31)
Age, years (SD)	32.4 (8.0)	30.1 (5.9)	31.2 (6.9)
Ethnicity, *n* (%)			
White British	13 (93)	16 (94)	29 (94)
Asian/Asian British—Pakistani	0 (0)	1 (6)	1 (3)
Asian/Asian British—other Asians	1 (7)	0 (0)	1 (3)
Number on acetazolamide (%)	4 (29)	6 (35)	10 (32)
Opening LP pressure, cmH_2_O (SD)	32.7 (4.8)	33.7 (6.3)	33.3 (5.6)
Weight, kg (SD)	108.4 (42.3)	97.9 (21.3)	102.6 (32.3)
BMI, weight (kg)/height (m^2^) (SD)	41.2 (16.6)	37.3 (7.2)	39.2 (12.6)
HIT-6 score (SD)	63.4 (8.1)	63.8 (8.2)	63.6 (8.0)
IIH symptoms, *n* (%)			
Headache	14 (100)	16 (94)	30 (97)
Visual loss	8 (57)	4 (24)	12 (39)
Pulsatile tinnitus	13 (93)	12 (71)	25 (81)
Diplopia	5 (36)	7 (41)	12 (39)
Transient visual obscurations	6 (43)	6 (35)	12 (39)
PMD, dB (SD)	−3.4 (6.8)	−6.1 (5.4)	−4.8 (6.1)
Log visual acuity (SD)	0.13 (0.22)	0.08 (0.23)	0.10 (0.22)
Log contrast sensitivity	*N* = 12	*N* = 13	*N* = 25
	1.63 (0.16)	1.63 (0.22)	1.63 (0.19)
OCT, thickness in μm (SD)	*N* = 10	*N* = 17	*N* = 27
Average retinal nerve fibre layer	158.4 (83.0)	152.0 (68.7)	154.4 (72.8)
Maximum retinal nerve fibre	290.0 (102.4)	320.2 (117.2)	309.6 (110.4)
Frisen grading, *n* (%)	*N* = 11	*N* = 16	*N* = 27
1	2 (18)	4 (25)	6 (22)
2	5 (45)	9 (56)	14 (52)
3	3 (27)	0 (0)	3 (11)
4	1 (9)	2 (13)	3 (11)
5	0 (0)	1 (6)	1 (4)

Visual data are from the worst eye only. BMI = body mass index; HIT-6 = headache impact test-6; LP = lumbar puncture; OCT = optical coherence tomography; SD = standard deviation.

### Clinical outcomes

#### Primary clinical outcome

**Figure 2:**
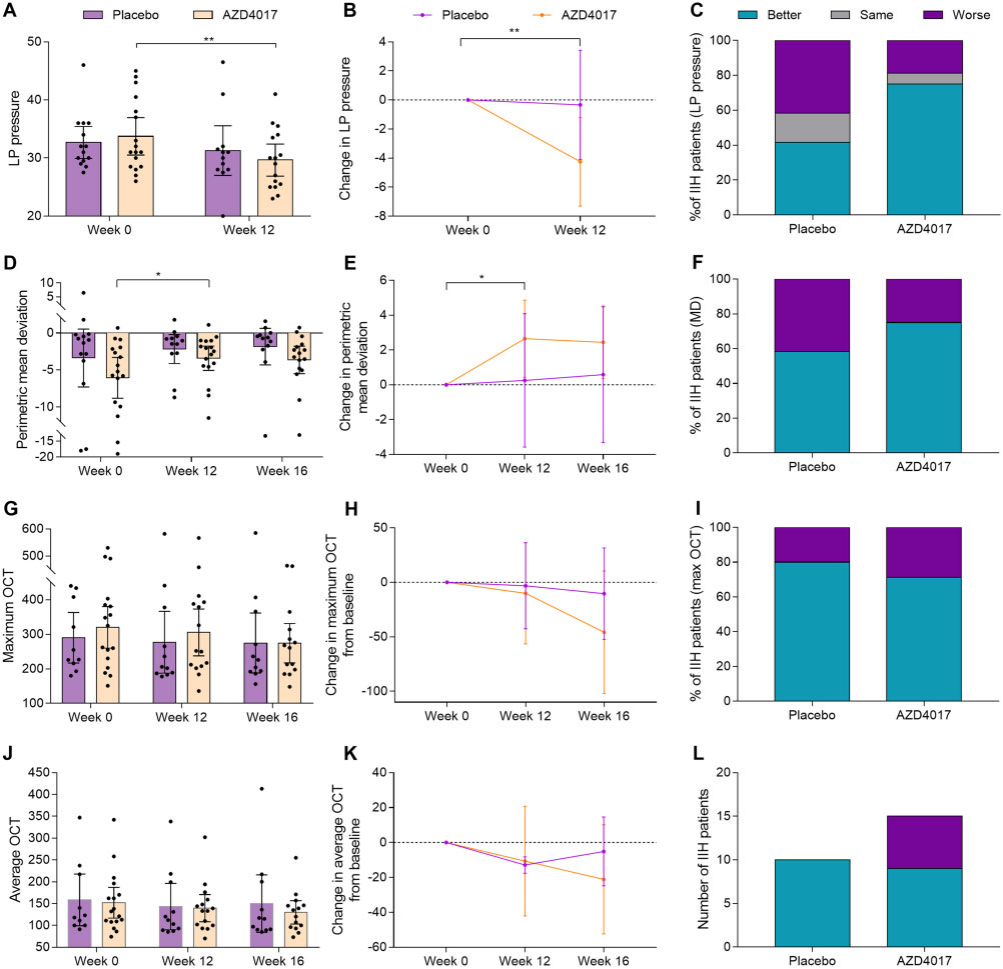
**Clinical outcomes following treatment with AZD4017 and placebo for 12 weeks and then 4 weeks after stopping treatment.** (**A**) Absolute LP pressure. (**B)** Change in LP pressure. (**C**) Percentage of patients with better, same or worse LP pressure at 12 weeks. **(D)** Absolute visual field mean deviation (dB). **(E)** Change in visual field mean deviation. (**F**) Percentage of patients with better, same or worse visual field mean deviation at 12 weeks. (**G**) Absolute maximum OCT RNFL height (µm). (**H**) Change in maximum OCT RNFL height. (**I**) Percentage of patients with better, same or worse maximum OCT RNFL height at 12 weeks. (**J**) Average OCT RNFL height (µm). (**K**) Change in average OCT RNFL height. (**L**) Number of patients with better, same or worse average OCT RNFL height at 12 weeks. The number of OCT scans performed varied during the conduct of the trial due to patients declining this aspect of the protocol and due to times when the scanner was not operating. Data are presented as mean ± 95% confidence index. *<0.05, **<0.01. LP = lumbar puncture, OCT = optical coherence tomography, RNFL = retinal nerve fibre layer

At 12 weeks, the mean ICP was 29.7 cmH_2_O (SD = 5.2) in the AZD4017 group compared with 31.3 cmH_2_O (SD = 6.7) in the placebo group [adjusted mean difference: −2.8 cmH_2_O, 95% confidence interval (CI): −7.1 to 1.5; *P* = 0.2; Fig. 2A]. An exploratory analysis assessed the mean change in ICP within each group. ICP decreased from 33.7 (SD = 6.3) at baseline to 29.7 cmH_2_O (SD = 5.2) at 12 weeks in the AZD4017 group [mean change: −4.3 cmH_2_O (SD = 5.7); *P* = 0.009] and from 32.7 (SD = 4.8) to 31.3 cmH_2_O (SD = 6.7) in the placebo group [mean change: −0.3 cmH_2_O (SD = 5.9); *P* = 0.8; Fig. 2B and C].

#### Secondary clinical outcomes

At Weeks 12 and 16, there were no statistically significant differences between the two treatment groups in IIH symptoms (Supplementary Table 2). At 12 and 16 weeks, the Humphrey Visual Field PMD (worst eye) was not significantly different between groups (adjusted mean difference at 12 weeks: 0.3 dB, 95% CI: −2.0 to 2.7, *P* = 0.8; Fig. 2D–F, Table 2 and Supplementary Table 3). However, within-group analysis showed that the PMD improved from −6.1 dB (SD = 5.4) at baseline to −3.4 dB (SD = 3.2) [mean change 2.7 dB (SD = 4.3), *P* = 0.04] at 12 weeks in the AZD4017 group and from −3.4 dB (SD = 6.8) to −2.2 dB (SD = 3.1) [mean change 0.3 dB (SD = 6.0), *P* = 1.0] in the placebo group. There were also no statistically significant differences between groups at either 12 or 16 weeks in visual acuity, contrast sensitivity, optical coherence tomography average and maximal retinal nerve fibre layer [Table 2; Fig. 2G–I (maximum retinal nerve fibre layer) and J–L (average retinal nerve fibre layer) and Supplementary Table 3]. At 12 weeks, the mean Frisen grade in the worst eye was 1.56 (SD = 0.96) in the AZD4017 group and 2.25 (SD = 0.87) in the placebo group (adjusted mean difference: −0.7, 95% CI: −1.4 to 0.3; *P* = 0.06).

**Table 2:** Visual function and optic nerve head at baseline and Week 12

Worse eye	Baseline, mean (SD)		Week 12, mean (SD)		Adjusted mean difference at 12 weeks (95% CI)	*P*-value
	Placebo	AZD4017	Placebo	AZD4017		
Visual acuity LogMAR	0.13 (0.22)	0.08 (0.23)	0.09 (0.18)	0.06 (0.15)	−0.03 (−0.12 to 0.07)	0.5
Contrast sensitivity	1.63 (0.16)	1.63 (0.22)	1.66 (0.12)	1.65 (0.15)	−0.02 (−0.15 to 0.11)	0.7
PMD	−3.4 (6.8)	−6.1 (5.4)	−2.2 (3.1)	−3.4 (3.2)	0.3 (−2.0 to 2.7)	0.8
OCT RNFL average (μm)	158.4 (83.0)	152.0 (68.7)	143.2 (78.7)	139.7 (56.3)	0.1 (−34.0 to 34.1)	1.0
OCT maximal RNFL (μm)	290.0 (102.4)	320.2 (117.2)	277.0 (133.1)	305.5 (122.3)	−4.5 (−68.1 to 59.1)	0.9
Average Frisen grading	2.27 (0.90)	2.19 (1.17)	2.25 (0.87)	1.56 (0.96)	−0.7 (−1.4 to 0.03)	0.06

All measures shown in the table are of worst eye. Negative values in the adjusted mean difference between treatment arms favour AZD4017. CI = confidence interval; LogMAR = log of the minimum angle of resolution; OCT = optical coherence tomography; RNFL = retinal nerve fibre layer.

Data from both eyes were also analysed but yielded equivalent results to that of the worst eye.

All headache outcomes were not statistically significantly different between AZD4017 and placebo at Weeks 12 or 16 (Supplementary Table 4). There were also no statistically significant differences in any of the anthropometric outcomes (body mass index, waist:hip ratio). Specifically, the mean difference in body mass index at 12 weeks between arms was 0.4 kg/m^2^ (95% CI: −0.6 to 1.4). Both trial arms saw a minimal increase in weight: AZD4017 group increased by 1.21 kg (95% CI: −0.47 to 2.89) and the placebo group increased by 0.04 kg (95% CI: −1.88 to 1.96). The body mass index change also saw a small increase in both groups: AZD4017 group by 0.6 kg/m^2^ (95% CI: −0.2 to 1.3) and placebo group by 0.2 kg/m^2^ (95% CI: −0.5 to 0.8).

### Safety and tolerability

Study medication was well tolerated with participants in both arms taking on average 98% of the total 168 study medication doses [mean doses taken were 164 (range 146–168) and 165 (range 158–168) in the AZD4017 and placebo groups, respectively]. There were no participant withdrawals due to adverse effects. Nine adverse events (in six participants) were deemed related to AZD4017, none were serious and three were due to non-clinically relevant fluctuations in serum cortisol. Adverse events are shown in Supplementary Table 5. One serious adverse event was reported in the placebo arm and deemed unrelated (fulminant deterioration in IIH necessitating CSF shunting 1 day post-randomization).

No differences were noted between treatment groups for the safety blood tests (Supplementary Table 6). As expected, there was a rise in the hypothalamic pituitary adrenal stimulatory hormone, adrenocorticotropic hormone, over 12 weeks in the AZD4017 group (mean difference at 12 weeks: 12.36 ng/l, 95% CI: −0.03 to 24.74). There was no difference in serum cortisol, testosterone or androstenedione, although serum dehydroepiandrosterone sulphate, a marker of adrenal androgen production, was higher at 12 weeks in the AZD4017 group (mean difference at 12 weeks: 5.44 nmol/l, 95% CI: 1.09–9.79); levels returned to normal 4 weeks after treatment cessation (Week 16).

### *In vivo* assessments

#### Blood and cerebrospinal fluid levels of AZD4017 and glucocorticoids

AZD4017 concentrations were detected in the serum after 1 week of treatment and sustained at Week 12 (*n* = 6). The presence of AZD4017 in the CSF was 0.5% that of the serum (Supplementary Table 7). No AZD4017 was detected in the placebo group at any time point.

**Figure 3:**
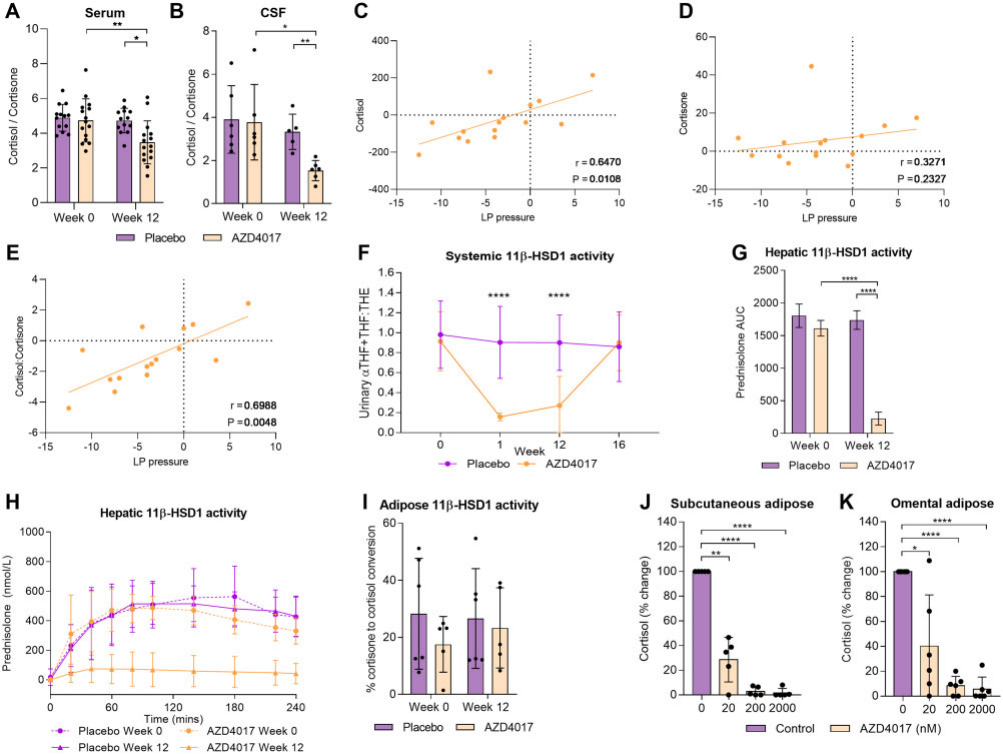
***In vivo* and *ex vivo* analyses of 11β-HSD activity after 12 weeks treatment with either AZD4017 or placebo.** (**A**) Serum cortisol:cortisone ratio. (**B**) CSF cortisol:cortisone ratio. (**C**) Urinary 11β-HSD1 activity [(5α-THF + THF):THE] at Weeks 0, 1, 12 and 16. (**D**) Change in prednisolone AUC (see **E**). (**E**) Hepatic 11β-HSD1 activity (mean blood prednisolone concentration after conversion from prednisone) over 4 hours. (**F**) Subcutaneous adipose 11β-HSD1 activity (percentage change from cortisone to cortisol) *ex vivo*. (**G**) *Ex vivo* subcutaneous adipose. (**H**) Omental adipose 11β-HSD1 activity (cortisol production from cortisone) after 24 hours incubation with 0, 20, 200 or 2000 nM of AZD4017 *in vitro.* Data presented as mean ± SD. *P < 0.05, **P < 0.01, 0.001, ****P < 0.0001. AUC = area under the curve; 5α-THF = 5α-tetrahydrocortisol; THE = tetrahydrocortisone; THF = tetrahydrocortisol.

Serum and CSF cortisol and cortisone were examined in the placebo and AZD4017 groups at baseline and at 12 weeks. There was no difference in the serum cortisol:cortisone ratio at baseline; however, the ratio fell significantly in the AZD4017 group between baseline and 12 weeks (*P* = 0.0083), while it did not in the placebo group, and was significantly lower in the AZD4017 group than in the placebo group at 12 weeks (*P* = 0.0125), indicating inhibition of 11β-HSD1 activity (Fig. 3A). Similarly, the CSF cortisol:cortisone ratio did not differ between arms at baseline; however, at Week 12, there was a significant decrease in the CSF cortisol:cortisone in the AZD4017 group compared with placebo (*P* = 0.002) and the AZD4017 group between baseline and 12 weeks (*P* = 0.03) (Fig. 3B), implying that the systemic inhibition of 11β-HSD1 activity can regulate CSF glucocorticoid exposure. Importantly, in the AZD4017 group, changes between baseline and 12 weeks (*n* = 15 with paired data) in both the serum cortisol and the cortisol:cortisone ratio significantly correlated with change in lumbar puncture pressure (*R* = 0.65, *P* = 0.01 and *R* = 0.70, *P* = 0.005, respectively), while, as expected, changes in the cortisone levels did not (Fig. 3C–E). There was no correlation between body mass index and the serum cortisol or cortisol:cortisone ratio. In the small subgroup (*n* = 6) in which changes in CSF glucocorticoids were measured, we did not identify a significant correlation with changes in lumbar puncture pressure.

#### *In vivo* systemic 11β-hydroxysteroid dehydrogenase activity

The urinary (5α-tetrahydrocortisol +tetrahydrocortisol):tetrahydrocortisone glucocorticoid metabolite ratio reflective of systemic 11β-HSD1 activity was significantly reduced in AZD4017 versus placebo groups at Week 1 (0.16 ± 0.04 versus 0.90 ± 0.36, *P* < 0.0001) and Week 12 (0.27 ± 0.29 versus 0.90 ± 0.28; *P* < 0.0001). In contrast, the ratios did not differ between the two treatment groups at baseline (*P* = 0.6) and 4 weeks after the end of treatment (Week 16, *P* = 0.8). 11β-HSD2 activity as assessed by urinary cortisol over cortisone remained unchanged and similar in both groups throughout the 12 weeks of treatment (*P* = 0.6). These data imply that AZD4017 was effective at inhibiting 11β-HSD1 (Fig. 3C). No correlation was found between the change in (5α-tetrahydrocortisol +tetrahydrocortisol):tetrahydrocortisone and ICP (*R* = 0.1; *P* = 0.7) or PMD (*R* = 0.2; *P* = 0.4).

#### Hepatic 11β-hydroxysteroid dehydrogenase activity

The placebo group had robust capacity to generate prednisolone following oral prednisone at both baseline and after 12 weeks. The baseline prednisolone generation curve for the AZD4017 group was indistinguishable from the placebo curve; however, at 12 weeks, the AZD4017 group was essentially unable to generate prednisolone (Fig. 3D and E), indicating effective inhibition of hepatic 11β-HSD1 activity. Area under the curve analysis of the mean time points at 12 weeks showed significantly impaired prednisolone generating capacity for AZD4017 versus placebo (228 ± 99 versus 1738 ± 142; *P *<* *0.0001), an 85.9% reduction (*P *<* *0.0001) in overall prednisolone generating capacity after 12 weeks (Fig. 3D and E). There was no correlation between the change in the area under the curve for prednisolone and ICP (*R *=* *0.1; *P *=* *0.8) or PMD (*R *=* *0.4; *P *=* *0.2).

#### Adipose 11β-hydroxysteroid dehydrogenase activity

While AZD4017 effectively inhibited hepatic 11β-HSD1, we were unable to show impaired capacity to generate cortisol from cortisone in explanted subcutaneous adipose tissue biopsies. At baseline and following 12 weeks of oral AZD4017 (*n* = 5), there was no significant change in total cortisol versus placebo (9.0 ± 5.6 versus 12.4 ± 4.9 nmol; *P *=* *0.3) or percentage conversion of cortisone to cortisol (23 ± 14 versus 27 ± 18%; *P *>* *0.99) and no change in those treated with placebo (*n* = 6; Fig. 3F). However, AZD4017 was able to significantly inhibit 11β-HSD1 activity when added to *ex vivo* adipose explants from subcutaneous and omental depots. The 20 nM AZD4017 significantly impaired the conversion of cortisone to cortisol (>70% versus control), and 200 nM onwards was sufficient to effectively block cortisol generation, particularly in the subcutaneous depot (Fig. 3G and H).

## Discussion

We report the first phase II RCT assessing an 11β-HSD1 inhibitor AZD4017 for the treatment of IIH. We have shown some possible clinical benefit for AZD4017 and have also shown that it was well tolerated and safe. We found evidence for effective *in vivo* 11β-HSD1 inhibition.

Our primary hypothesis stated that 11β-HSD1 inhibition in patients with IIH would reduce CSF secretion and lower ICP while being safe and tolerable following 12 weeks of treatment. ICP was the primary clinical outcome measure, representing the hallmark of the disease driving clinical sequelae. At 12 weeks, although ICP was lower in the AZD4017 group compared with placebo, the difference between groups was not statistically significant. Exploratory analyses of the mean change within groups found a significant improvement in ICP in the AZD4017 group between baseline and 12 weeks but not in the placebo group. In support of our hypothesis, among the AZD4017 group, the change in serum cortisol and cortisol:cortisone ratio over the treatment period, a marker of 11β-HSD1 inhibition, correlated significantly with reduction in ICP. Of note, a minimal clinically important change in ICP in IIH has not been determined in IIH and establishing one would be useful for future trials. In addition, previous trials have noted that ICP reduction below the cut-off of 25 cmH_2_O is not universally required to translate into resolution of IIH clinical features (Sinclair *et al.*, 2010*a*).

The visual field perimetric assessment is another clinically meaningful measure and has been selected as the primary outcome measures in previous IIH trials. We found no difference between groups in PMD at 12 weeks; however, there was significant improvement over time in the AZD4017 arm but not in the placebo arm. This may reflect the pragmatic recruitment of all degrees of PMD at enrolment (including those with severe visual loss with limited capacity to improve), while other trials have restricted enrolment to a selected cohort (e.g. −2 to −5 dB) (NORDIC Idiopathic Intracranial Hypertension Study Group Writing Committee *et al.*, 2014). In addition, in those with a PMD near normal at baseline (despite papilloedema), there may be a floor effect, where no further improvement is possible in the PMD in these individuals. In addition, this small trial was not powered to determine significance in the secondary outcome measures.

Headache is a key disabling feature in IIH (Mulla *et al.*, 2015). We did not detect differences between the groups in any of the headache assessments at 12 weeks, although data from the patient-completed headache impact test-6 favoured the AZD4017 group. Evaluating the effect of AZD4017 on headache measures over a longer treatment duration would be of interest.

Previous trials showed that 11β-HSD1 inhibition leads to adaptive changes in hypothalamic pituitary adrenal stimulatory hormone adrenocorticotropic hormone and the adrenal androgen precursor dehydroepiandrosterone sulphate. Our data support these findings, but with no significant change in downstream effector hormones (cortisol and testosterone).

*In vivo* evaluation of our patients demonstrated that AZD4017 was a highly effective systemic and hepatic 11β-HSD1 inhibitor, in line with previous studies using 11β-HSD1 inhibitors in humans (Courtney *et al.*, 2008; Schwab *et al.*, 2017). Systemic efficacy may modify metabolic aspects of IIH with indirect benefits on ICP (Hornby *et al.*, 2018).

While AZD4017 effectively inhibited 11β-HSD1 when applied to subcutaneous and omental adipose tissue explants, we were unable to prove inhibition *in vivo* and propose that with this experimental design, 11β-HSD1 activity recovers over the assay period once removed from AZD4017, a reversible competitive inhibitor.

Blood–brain barrier AZD4017 penetrance was low, with levels in the CSF 0.5% those of plasma levels, but was associated with reduced CSF cortisol:cortisone ratio suggesting that 11β-HSD1 may contribute to cortisol availability in the CSF.

### Limitations

We were unable to directly evaluate 11β-HSD1 inhibition at the choroid plexus, the tissue responsible for CSF secretion; hence, we cannot be certain of inhibition by 11B-HSD1 at the target tissue. We have evaluated the efficacy of other IIH drugs using rodent ICP monitoring models (Botfield *et al.*, 2017; Scotton *et al.*, 2019), but AZD4017 is only effective in humans and primates, thus limiting our ability to evaluate its action in rodent models. The trial duration was likely too short, with insufficient time to detect clinical efficacy. A duration of 12 weeks was chosen for the evaluation of safety and tolerability and represented the longest duration of dosing to date with AZD4017. This may not have been sufficient for the meaningful evaluation of clinical outcomes with other IIH RCTs evaluating drugs over a 6-month period (Committee *et al.*, 2014). The enrolment criteria for the study were deliberately broad allowing inclusion of a spectrum of patients with IIH with active disease and ensuring the generalizability of results; however, this did not allow evaluation in disease subgroups such as those with mild visual loss versus those with severe irreversible visual loss. Finally, the sample size (31 participants) is small, which may have reduced our power and limited meaningful evaluation of clinical measures and the trial was not designed to establish significant changes in the secondary clinical outcome measures.

## Conclusion

This is the first phase II study evaluating the novel pharmacological therapy AZD4017 in IIH. We demonstrate safety, tolerability and provide strong *in vivo* evidence for effective 11β-HSD1 inhibition. There was a significant reduction in ICP in the AZD4017 and not the placebo group over the treatment duration (exploratory within-group analysis) and reduction in ICP significantly correlated with reduction in serum cortisol:cortisone ratio; however, the primary analysis evaluating the difference between groups at 12 weeks did not reach statistical significance. The data suggest that 11β-HSD1 inhibition may have utility for reducing the effects and consequences of raised ICP in patients with IIH. Further evaluation of these therapeutic strategies in this disabling disease, for which few useful medical options exist, would be worthwhile.

## Supplementary Material

fcz050_Supplementary_Data

## Abbreviations

11β-HSD1 =: 11β-hydroxysteroid dehydrogenase type 1
CSF =: cerebrospinal fluid
ICP =: intracranial pressure
IIH =: idiopathic intracranial hypertension
LC–MS/MS =: liquid chromatography–tandem mass spectrometry
PMD =: perimetric mean deviation
RCT =: randomized controlled trial
