# Corrigendum to: 11β-Hydroxysteroid dehydrogenase type 1 inhibition in idiopathic intracranial hypertension: a double-blind randomized controlled tria

**DOI:** 10.1093/braincomms/fcaa108

**Published:** 2020-08-25

Keira Markey, James Mitchell, Hannah Botfield, Ryan S. Ottridge, Tim Matthews, Anita Krishnan, Rebecca Woolley, Connar Westgate, Andreas Yiangou, Zerin Alimajstorovic, Pushkar Shah, Caroline Rick, Natalie Ives, Angela E. Taylor, Lorna C. Gilligan, Carl Jenkinson, Wiebke Arlt, William Scotton, Rebecca J. Fairclough, Rishi Singhal, Paul M. Stewart, Jeremy W. Tomlinson, Gareth G. Lavery, Susan P. Mollan and Alexandra J. Sinclair. 11β-Hydroxysteroid dehydrogenase type 1 inhibition in idiopathic intracranial hypertension: a double-blind randomized controlled trial. Brain Communications 2020. doi: 10.1093/braincomms/fcz050.

In the above article co-author Rebecca J Fairclough’s name was initially misspelled. This has now been corrected.

