# Supplementary material for: 11β-Hydroxysteroid dehydrogenase type 1 inhibition in idiopathic intracranial hypertension: a double-blind randomized controlled trial
Source: Brain Commun. 2020 Jan 10;2(1):fcz050. doi: 10.1093/braincomms/fcz050 (PMC7425517; doi:10.1093/braincomms/fcz050)

Lowering Intracranial Pressure in Idiopathic Intracranial Hypertension: Assessing the therapeutic efficacy and safety of an 11β-hydroxysteroid dehydrogenase type 1 inhibitor (AZD4017): the IIH:DT Trial

Trial registration number: ISCRCTN 2013-003643-31

Statistical Analysis Plan

Version Number	Effective Date	Protocol Version Number
2.0	18th September 2017	8.0

**This Statistical Analysis Plan has been approved by:**					
Author:	Rebecca Woolley	Signature:		Date:	
Reviewer:	Natalie Ives	Signature:		Date:	
Chief Investigator:	Alex Sinclair	Signature:		Date:	
Name of Blind Reviewer (if required; otherwise insert N/A):	N/A	Signature:	-	Date:	-

Version number	Effective date	Protocol version number	Details of changes (refer to section number of SAP)	Reason for change	Timing of change with respect to interim / final analysis
1.0	05/05/17	8.0	First release	-	-
2.0		8.0	Minor edits to clinical information in background and units for lumbar puncture (throughout SAP).<br>Section 6.2: Clarified data to be used from headache diary (headache frequency, severity and duration). Added further information on the assessment of the Fundus photographs by masked neuro-ophthalmologist.<br>Section 9.12: Added standard definitions for how severity and duration of headache calculated in headache trials. Added how the severity scores from v1 of headache diary will be mapped to scores on v2 of headache diary. Defined worst and best eye.<br>Section 10.6: Analysis of individual eye will now be based on worse and best eye, rather than left and right eye.<br>Section 10.6: Analysis of Fundus photograph data clarified.<br>Section 10.10: Analysis now purely descriptive. List of safety data items extended. Data to be analysed	Clinical clarification. Lumbar puncture unit cmCSF rather than cmH_20.<br>Missing information from SAP v1.0.<br>Missing information from SAP v1.0.<br>Analysis in line with current practice, and how a recent trial reported their individual eye data<br>Had clarification from the Chief Investigator as to how this data should be analysed, which is also as per a recent trial.<br>Chief Investigator clarified safety data items they wished to be analysed, and to report means	Prior to database lock and final analysis

			simply as means at each time point, and proportion above upper or trial upper limit as appropriate.	and proportion above upper reference limit or upper trial limit as appropriate.	

**Abbreviations & Definitions**	
**Abbreviation / Acronym**	**Meaning**
AE	Adverse Event
BCTU	Birmingham Clinical Trials Unit
BMI	Body Mass Index
DMC	Data Monitoring Committee
CMO	Contract Manufacturing Organisation
CONSORT	Consolidated Standards of Reporting Trials
HIT 6	Headache impact test-6
ICP	Intracranial pressure
IIH	Idiopathic intracranial hypertension
ISCRCTN	International Standard Randomised Controlled Trial Number
OCT	Optical coherence tomography
SAE	Serious Adverse Event
SAP	Statistical Analysis Plan
TSC	Trial Steering Committee

Contents

1. Introduction 6
2. Background and rationale 6
3. Study objectives 6
4. Study design 6
5. Study comparisons 6
6. Outcome measures 6
6.1. Primary outcome(s) 6
6.2. Secondary outcomes 7
7. Randomisation 8
8. Sample size 8
9. General statistical considerations 8
9.2. Definition of compliance 9
9.3 Handling of protocol deviations and violations 9
9.4. Levels of confidence and p-values 9
9.5. Adjustments for multiplicity 9
9.6. Interim analysis and stopping rules 9
9.7. Timing of primary analysis 10
9.8. Timing of other analyses 10
9.9. Covariate adjustment 10
9.10. Handling missing data 10
9.11. Distributional assumptions and outlying responses 11
10. Proposed statistical methods 12
10.1. Study population 12
10.2. Baseline characteristics 12
10.3. Compliance to allocation 13
10.4. Protocol deviations and violations 13
10.5. Analysis methods – primary outcome 13
10.6. Analysis methods – secondary outcomes 13
10.7. Analysis methods – exploratory and other outcomes 14
10.8. Sensitivity analyses 14
10.9. Planned subgroup analyses 14
10.10. Safety data 14
11. Output from sub-randomisations 14
12. Health economic analyses 15
13. Statistical software 15
14. References 15
Appendix A – Deviations from SAP 16
Appendix B – Trial schema 16
Appendix C – Schedule of assessments 17
Appendix D – CONSORT flow diagram 18

1. Introduction

This document gives a detailed statistical analysis plan for the IIH:DT trial and should be read in conjunction with the current trial protocol. Any deviations from this plan will be described in the final report or publication (see Appendix A).

2. Background and rationale

The background and rationale for the trial are outlined in detail in the protocol. In brief, IIH:DT is a trial in participants who have idiopathic intracranial hypertension (IIH) which is characterised by elevated intracranial pressure (ICP) and papilloedema. IIH is a condition found almost exclusively in obese women (90%) and causes disabling daily headaches and loss of vision, which is severe and permanent in up to 25% of cases. Is it hypothesised that 11β-HSD1 amplifies local cortisol availability at the choroid plexus, thereby increasing cerebrospinal fluid secretion which may increase ICP. Therefore use of an 11β-HSD1 inhibitor may lead to decreasing ICP in IIH.

3. Study objectives

The primary objective is to evaluate whether 12 weeks of treatment with the 11β-HSD1 inhibitor, AZD4017, is a potential treatment for IIH through reduction in ICP.

Secondary objectives are as follows:

- Evaluate the effects of AZD4017 on vision, papilloedema, headache and anthropological measurements (including weight and fat mass distribution) in participants with IIH;
- Evaluate the safety and tolerability of a 12 week course of AZD4017 in participants with IIH.

4. Study design

IIH:DT is a multicentre, prospective, double-blind, placebo-controlled, parallel group, phase II, randomised controlled trial (see Appendix B for trial schema). Participants will be recruited from secondary care from Neuro-ophthalmology or neurology clinics. Participants will be randomised in a 1:1 ratio to either AZD4017 or placebo. Participants will receive either AZD4017 or placebo twice daily for 12 weeks.

5. Study comparisons

All references in this document to ‘group’ refer to AZD4017 or placebo.

6. Outcome measures

6.1. Primary outcome(s)

The primary outcome is to examine the effect of AZD4017 on ICP, as measured by lumbar puncture in cmCSF, from baseline to 12 week. The primary outcome measure is the difference in ICP at 12 weeks.

6.2. Secondary outcomes

Secondary outcomes are as follows:

- IIH symptoms (presence or absence of tinnitus, perceived visual loss, diplopia, visual obscuration and headache) over 12 weeks, and at 16 weeks;
- IIH visual function in both eyes (measured by LogMAR (log of the minimum angle of resolution) chart to assess visual acuity, automated perimetry (Humphrey 24-2 central threshold) to measure the visual field mean deviation and MARs charts to evaluate contrast sensitivity) between baseline, week 12 and week 16;
- Papilloedema evaluated using 1). spectral domain optical coherence tomography and 2). Fundus photographs with Frisen classification (by masked neuro-ophthalmologists to grade the images and who also assess whether the image is better, worse or the same as the image taken at baseline) between baseline, week 12 and week 16;
- Headache associated disability using the headache impact test-6 score (HIT 6), headache diary (to assess headache frequency (days/week), severity and duration) and use of analgesia (days/week) between baseline and 12 weeks, with further analysis between weeks 12 and 16;
- Anthropological measures (e.g. blood pressure, body mass index (BMI), waist/hip ratio and fat distribution);
- Safety and tolerability of AZD4017 measured using adverse event (AE) and serious adverse event (SAE) reporting and safety bloods.

Fundus photographs will be reviewed by three masked neuro-ophthalmologists who will grade the images as described above. The overall grading of an image will be where the majority of the neuro-ophthalmologists are in agreement on a grade, or in the instance where the neuro-ophthalmologists are in disagreement by at most one grade, the median will be taken as the grade. If the neuro-ophthalmologists disagree by more than one grade, these images will be checked by a 4th neuro-ophthalmologist to obtain a consensus grade.

The HIT 6 is a patient completed questionnaire where a score of 36=best outcome, and a score of 78=worst outcome.

The Headache Diary is completed over 7 days at baseline (participants complete daily headache diary in week before screening visit) and weeks 12 and 16 (participants complete daily headache dairy in week prior to visit). Severity (0-5)* and duration (over each 24 hour period) of headache are reported for each day, along with use of painkillers.

Secondary outcomes will be assessed at weeks 12 and 16 (see Appendix C for a table of assessment times). The safety bloods are also collected at the week 1, week 4 and week 8 time points.

Adverse events and SAEs are collected as and when they occur over the course of the 16 week

follow up.

*updated version of the headache diary scores severity 0-10 (see section 9.12).

7. Randomisation

Randomisation will be performed by the Contracting Manufacturing Organisation (CMO), Almac, who are supplying and coding the study medications on behalf of AstraZeneca. Block randomisation will be used so that each block of trial numbers contains a random assignment of equal numbers of active and placebo treatment allocations. Participants will be randomised in a 1:1 ratio to receive either AZD4017 or placebo.

Tablets containing either AZD4017 or matching placebo will be prepared by Almac appointed by AstraZeneca.

8. Sample size

A total of 30 participants will be recruited, 15 participants in each group (AZD4017 vs. placebo).

To detect a difference between the groups of 14% in ICP (assuming a standard deviation of 10% for ICP) with 90% power and two-sided $\alpha=0.05$, requires 12 participants per group. Allowing for 20% drop out, 15 participants are required per group, so 30 participants in total.

9. General statistical considerations

9.1. Analysis populations

All primary analyses (primary and secondary outcomes) will be by intention-to-treat. Patients will be analysed in the treatment group to which they were randomised, and all patients shall be included whether or not they received the allocated treatment. This is to avoid any potential bias in the analysis.

The safety analysis set will include all subjects who received at least 1 dose of randomised study medication and for whom any post-dose data are available.

A ‘per protocol’ analysis (defined in section 9.2) for the primary outcome will also be carried out but only as a sensitivity analysis.

9.2. Definition of compliance

Compliance to treatment allocation will be monitored by counting the number of tablets that the participant has taken over the 12 week treatment period. Patients are randomised to receive 2x200mg of either AZD4017 or placebo twice daily for 12 weeks. Doses will be taken 12 hours apart, though this will not be monitored. The total number of tablets that a participant should have taken over the 12 week treatment period is 336 tablets. We will define treatment compliance as patients who took at least 80% of the total number of tablets (i.e. 268 tablets). For the per protocol analysis only those patients who took at least 80% of the tablets will be included in the analysis. Missed doses will be documented using both reported missed doses and tablet count (if available).

9.3 Handling of protocol deviations and violations

We will apply a strict definition of the intention-to-treat-principle and will consider all randomised patients in the analysis in some form regardless of deviation from the protocol.[1] This includes patients who were randomised but later found to violate the inclusion or exclusion criteria. It does not include those participants who have specifically requested to withdraw the use of their follow-up data in the first instance; however these outcomes will be explored as per other missing responses.

Data collected outside the scheduled window frame (+/- 1 weeks) for all assessment points will be included in the intention to treat analysis, but a sensitivity analysis will also be performed where those patients who returned the forms outside of the assessment window are excluded (see section 10.8 for more details on sensitivity analyses).

9.4. Levels of confidence and p-values

Unless otherwise specified, estimates of differences between groups will be presented with 95%, two-sided confidence intervals. P-values will be reported from two-sided tests at the 5% significance level.

9.5. Adjustments for multiplicity

No correction for multiple testing will be made.

9.6. Interim analysis and stopping rules

If AZD4017 is overwhelmingly better or worse than placebo with respect to ICP, then this effect may become apparent before the target recruitment has been reached. Alternatively, new evidence could emerge from other sources to suggest that AZD4017 is definitely more, or less, effective than placebo. To protect against any unnecessary continuance of the trial in this event, interim analyses of major endpoints and safety data will be supplied during the period of recruitment to the study, in strict confidence, to the Data Monitoring Committee (DMC) along with updates on results of other related studies, and any other analyses that the DMC may request.

The DMC will advise the chair of the Trial Steering Committee (TSC) if, in their view, any of the randomised comparisons in the trial have provided both: a) proof beyond reasonable doubt that for

all, or for some, types of participant one particular intervention is definitely indicated or definitely contra-indicated in terms of a net difference of a major endpoint, and b) evidence that might reasonably be expected to influence the patient management of many clinicians who are already aware of the other main trial results. Unless this happens, however, the TSC, the collaborators and all of the central Trial staff (except the statisticians who supply the confidential analyses) will remain ignorant of the interim results.

Appropriate criteria of proof beyond reasonable doubt cannot be specified precisely, but a difference of at least $p<0.001$ (similar to a Haybittle-Peto[2] stopping boundary) in an interim analysis of a major endpoint may be required to justify halting, or modifying, the study prematurely. If this criterion were to be adopted, it would have the practical advantage that the exact number of interim analyses would be of little importance, so no fixed schedule is proposed. Given the proposed use of the Haybittle-Peto boundary no adjustment for multiple testing (to control the overall type I error rate) is proposed, i.e. the threshold for statistical significance at final analysis will still be $p=0.05$.

A separate DMC reporting template will be drafted and agreed by the DMC including an agreement on which outcomes will be reported at interim analyses. The statistical methods stated in this Statistical Analysis Plan will be followed for the agreed outcomes.

9.7. Timing of primary analysis

The primary analysis for the study will occur once all participants have completed the 16 week assessment and the corresponding outcome data has been entered onto the study database and validated as being ready for analysis. This is provided the study has not stopped recruitment early for any reason (e.g. DMC advice or funding body request); if this is the case the analysis will be completed once the existing randomised participants have completed the 16 weeks assessment.

9.8. Timing of other analyses

N/A

9.9. Covariate adjustment

In the first instance, comparative estimates of differences between groups will be adjusted for the baseline values of the outcome variable of interest where available. If there are any imbalances at baseline in other important variables e.g. acetazolamide use, then these will also be included in the regression model as covariates.

9.10. Handling missing data

In the first instance, analysis will be completed on received data only with every effort made to follow-up participants even after protocol violation to minimise any potential bias. Participants will naturally be excluded from the primary analyses if they have no data available at the relevant assessment times or they withdraw/are withdrawn from the trial prior to the primary assessment

time. To examine the possible impact of missing data on the results, and to make sure we are complying with the intention-to-treat principle, sensitivity analysis will be performed on the primary outcome measure.[3] See section 10.8 for further details.

9.11. Distributional assumptions and outlying responses

Distributional assumptions (e.g. normality of regression residuals for continuous outcomes) will be assessed visually prior to analysis; although in the first instance the proposed primary method of estimation in this analysis plan will be followed. If responses are considered to be particularly skewed the impact of this will be examined through sensitivity analysis; this will consist of transformation of responses prior to analysis (e.g. log transformation) in the first instance. If extreme values are apparent and considered to be affecting the integrity of the analysis, sensitivity analysis consisting of removing the outlying response(s) and repeating the base analysis will be performed. Output from these analyses, if performed, will be described and presented alongside the base case analysis (or included, e.g. in appendices) with the excluded values clearly labelled. See section 10.8 for further details.

9.12. Data manipulations

The Trial Statistician will derive all responses from the raw data recorded in the database. The specifics of the data manipulations required are as follows.

Scoring for the following questionnaire responses will be calculated as per the following (refer to references for details):

- HIT-6: a six-item questionnaire with the following choice of responses: Never=6; Rarely=8; Sometimes=10; Very Often=11; and Always=13. Response scores from each of the questions are summed to produce the disability score. Score of 36=best outcome, and a score of 78=worst outcome;
- Headache Diary: diary is completed over 7 days prior to visit with information on severity and duration of headache, and use of painkillers. The total severity score is the severity summed up over the week divided by the number of days where headache was recorded, and the total duration score is the duration summed up over the week divided by the number of days where headache was recorded. If either severity or duration is missing, it is considered to be zero severity or duration for that day. The headache frequency is just the summation of the number of days a headache is experienced that week. In line with international Headache society reporting guidelines on headache outcomes which recommends reporting headache frequency per month, the weekly headache frequency will then be multiplied by 4 to provide a headache frequency per month (which will then be comparable with other trial datasets). Similarly, analgesic use will be calculated by summing up the number of times they are used over a week, then multiplied by 4 to provide analgesic use per month.

There are 2 versions of the headache diary. The first scored severity on a 0-5 scale. The second on a 0-10 scale. To combine the scores, the first version 0-5 scores will be mapped onto the second version scores using the following method: 0=0; 1=2; 2=4; 3=6; 4=8; and 5=10.

- Frisen Grading is scored on a 0-5 integer scale with 0=normal optic disk and 5=severe papilloedema.

Other outcomes will be calculated as follows:

- Age – number of days from date of birth to randomisation date divided by 365.25 to give age in years;
- Duration of IIH – number of days from date of diagnosis of IIH to randomisation date divided by 30.4 to give duration of IIH in months;
- Worst eye – the eye that has the worst Mean Deviation score on the Humphrey Visual field assessment at baseline will be considered the 'worst eye' for all analyses;
- Best (fellow) eye – the eye that has the better Mean Deviation score on the Humphrey Visual field assessment at baseline will be considered the 'best eye' for all analyses.

10. Proposed statistical methods

10.1. Study population

A flow diagram (recommended by CONSORT[4]) will be produced to describe the patient flow through each stage of the study. A template for reporting is given in Appendix D.

Numbers and description of reasons (where available) will be produced at each stage, e.g. reasons for withdrawing from the trial or why patients did not receive the allocated treatment. The following items (numbers of patients) will be described:

- Assessed for eligibility
- Eligible
- Randomised (by arm)
- Received allocated treatment/did not receive allocated treatment (by arm)
- Withdrawn/lost to follow up (by arm)
- Included in the primary analysis (by arm)

10.2. Baseline characteristics

The study population will be tabulated. Demographic variables will be described for the total population and for the two randomised arms separately. This will include age (years); ethnicity; BMI (kg/m^2); Weight (kg); presence of IIH symptoms; whether or not the participant was taking acetazolamide; opening ICP (cmCSF); HIT-6 score; and Frisen grading.

Categorical data will be summarised by number of responses, frequencies and percentages.

Continuous data will be summarised by the number of responses, mean and standard deviation if deemed to be normally distributed and number of responses, median and interquartile range if data appear skewed. Tests of statistical significance will not be undertaken.[5]

10.3. Compliance to allocation

A cross-tabulation of allocated treatment by the compliance categories stated in section 9.2 will be produced (proportions and percentages). The mean number of doses and the mean percentage of total dose will also be reported.

10.4. Protocol deviations and violations

Frequencies and percentages by group will be tabulated for the protocol deviations.

10.5. Analysis methods – primary outcome

The primary outcome is ICP at 12 weeks. ICP is measured by lumbar puncture in cmCSF. The number of observations, mean and standard deviation of the ICP at baseline and 12 weeks by treatment arm will be presented. A linear regression model will be used to compare the ICP at 12 weeks between the two arms, adjusting for baseline ICP. See 9.9 for more details on covariate inclusion.

10.6. Analysis methods – secondary outcomes

Continuous data items (e.g. HIT 6) will be analysed in the same way as the primary outcome. Analyses will be performed on data at 12 and 16 weeks. For the visual function data which is collected in both eyes, it is expected that the participant's data will be correlated, so the primary analysis will use data from both eyes, and will be analysed using a linear mixed model, with participant included as a random effect. The visual function and papilloedema data for worst eye and best eye will also be analysed separately as per the primary outcome, but this will be a secondary analysis.

The IIH symptom data is binary. The number and percentage of those with each symptom present will be presented at baseline and 12 and 16 weeks by treatment arm. Log-binomial models will be used to compare the symptom data at 12 and 16 weeks between the two arms, with baseline symptom included in the model as a covariate.

The Fundus photographs are given a Frisen Grading (score of 0-5) and the photographs at 12 and 16 weeks are compared to the baseline photograph to assess whether the image is better, worse or the same as the one taken at baseline (see section 6.2). These data items will be presented as categorical data items at each time point. A mean Frisen Grading will also be reported, and the data analysed as per the primary outcome. The same, better or worse data will be analysed at 12 and 16 weeks using a chi-squared test.

10.7. Analysis methods – exploratory and other outcomes

As indicated in the protocol there are a number of exploratory outcomes within the IIH:DT trial, the details of these analyses are documented separately, as they are not being undertaken within the Birmingham Clinical Trials Unit (BCTU).

10.8. Sensitivity analyses

Sensitivity analyses will be limited to the primary outcome and will consist of:

- Per-protocol analysis (population described in section 9.2);
- An analysis to assess the effect of missing responses using multiple imputation and last observation carried forward (the primary outcome is only collected at baseline and 12 weeks, so this is carrying forward the baseline ICP to 12 weeks, which is essentially assuming no change);
- An analysis to assess the effect of late assessments by including only those returned within the assessment window in the analysis.
- An analysis to assess the effect of any distributional assumptions (details provided in section 9.11);
- An analysis to assess the effect of any outliers.

10.9. Planned subgroup analyses

There are no planned subgroups analyses for this trial.

10.10. Safety data

No formal hypothesis testing of safety data is planned, although appropriate statistical techniques may be used to better understand the emerging safety profile if necessary.

The AE data will be summarised descriptively, as the number of AEs reported, and the number and percentage of patients reporting an AE by system organ class by treatment arm. The SAEs will also be summarised descriptively and tabulated as the number and percentage of patients experiencing an SAE by treatment arm. Information on relatedness to treatment will also be included for both AEs and SAEs.

Safety bloods are collected at: baseline; week 1; week 4; week 8; week 12; and week 16. Safety data on renal function (urea, creatinine, potassium, sodium), liver function (AST, ALT, ALP, bilirubin, albumin, yGT), thyroid function (TSH, fT4), muscle function (creatine kinase) and HPA (cortisol, DHEA, androstenedione, testosterone, ACTH, FSH, LH, oestradiol, progesterone) will be reported. For each measure, the mean and 95% confidence interval at each point by treatment arm will be calculated. The proportion of patients who has values above the upper reference limit or above the trial upper limit (e.g. AST, ALT, bilirubin) will be calculated.

11. Output from sub-randomisations

Not applicable.

12. Health economic analyses

No health economic analysis is planned for this study.

13. Statistical software

SAS software, version 9.4 (or higher) and/or Stata version 14 (or higher) will be used for all analyses.

14. References

1. Gupta SK. Intention-to-treat concept: A review. Perspect Clin Res. 2011; 2(3): 109-112.
2. Peto R, Pike MC, Armitage P, Breslow NE, Cox DR, Howard SV et al. Design and analysis of randomised clinical trials requiring prolonged observation of each patient. I. Introduction and design. Br J Cancer. 1976;34:585-612.
3. White IR, Horton NJ, Carpenter J, Pocock SJ. Strategy for intention to treat analysis in randomised trials with missing outcome data. BMJ. 2011; 342:d40.
4. Schulz KF, Altman DG, Moher D, for the CONSORT Group. CONSORT 2010 Statement: updated guidelines for reporting parallel group randomised trials. BMJ. 2010; 340:c332.
5. Altman DG, Dore CJ. Randomisation and baseline comparisons in clinical trials. Lancet. 1990; 335:149–153.

Appendix A – Deviations from SAP

This report below follows the statistical analysis plan dated <insert effective date of latest SAP> apart from following:

Section of report not following SAP	Reason
<insert section >	<insert, e.g. exploratory analyses request by TMG>

Appendix B – Trial schema

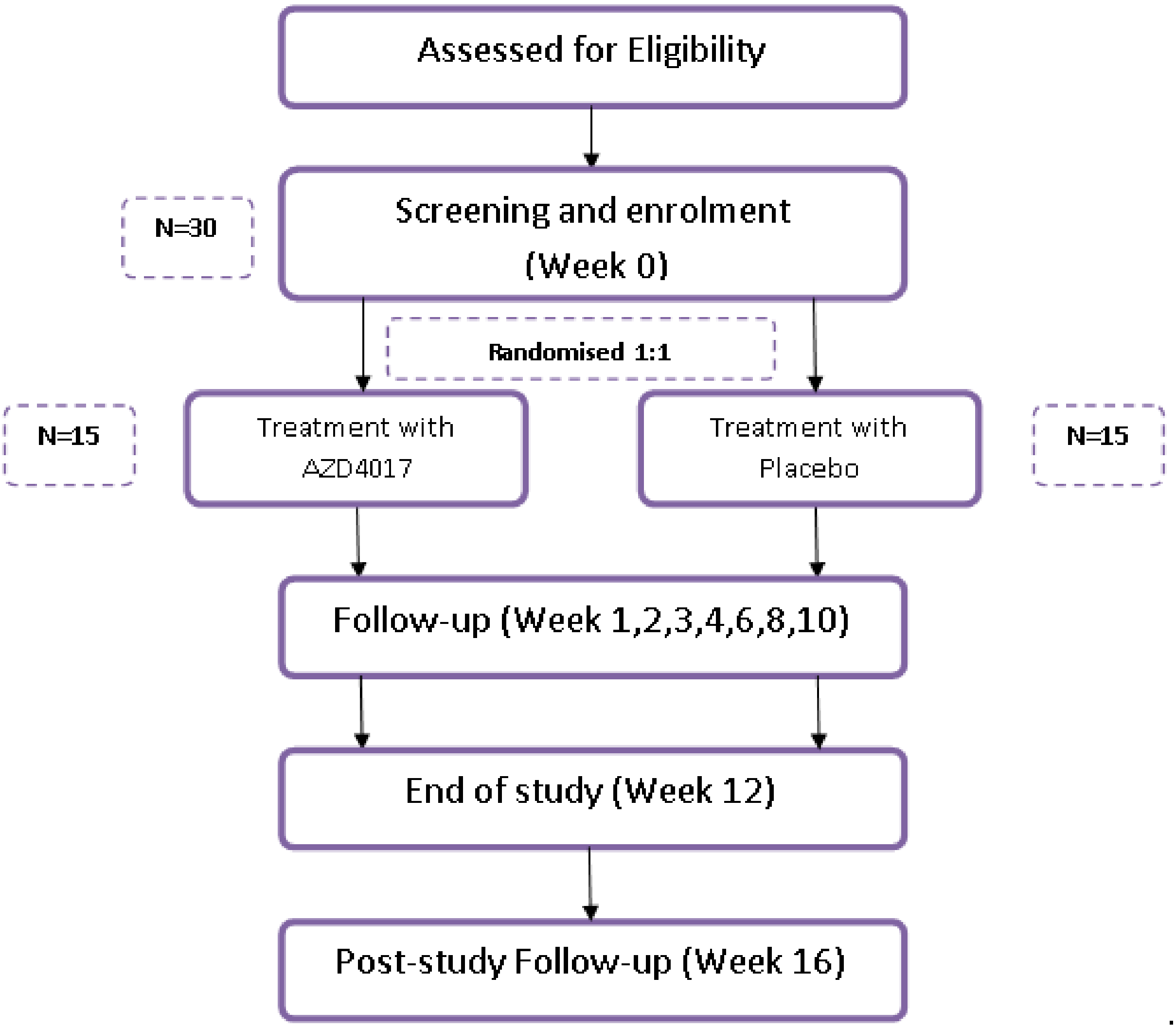

Appendix C – Schedule of assessments

		Screening	0	1	2	3	4	6	8	10	12 - D1	12- D2	16
**Primary outcome** ICP	Lumbar puncture & CSF sampling	■									■		
**Secondary outcomes** Weight	BMI, waist/hip ratio,	■									■		■
	DXA scanning		■									■	
	Blood pressure	■									■		
**Visual assessments**	Visual acuity and contrast sensitivity	■									■		■
	Humphrey visual field mean deviation	■									■		■
	Optical coherence tomography	■									■		■
	Retinal photographs (Frisen grade and "Better, worse, same evaluation"	■									■		■
**Headache**	Headache impact test 6	■									■		■
	Headache diary (frequency (days / weekly), severity & duration)	■									■		■
	Analgesic use (days/week)	■									■		■
**Metabolic evaluation**	24 hours urine sampling	■		■			■		■		■		■
	Fat biopsy		■									■	
	Prednisolone generation curve		■									■	
	DXA scanning		■									■	
**Medical assessment**	History, +/-examination, compliance	■		■	■	■	■	■	■	■	■		■
	Venesection		■	■			■		■			■	■
	Pregnancy test	■		■			■		■		■		■
**Serious adverse events monitoring**				■	■	■	■	■	■	■	■		■

Appendix D – CONSORT flow diagram

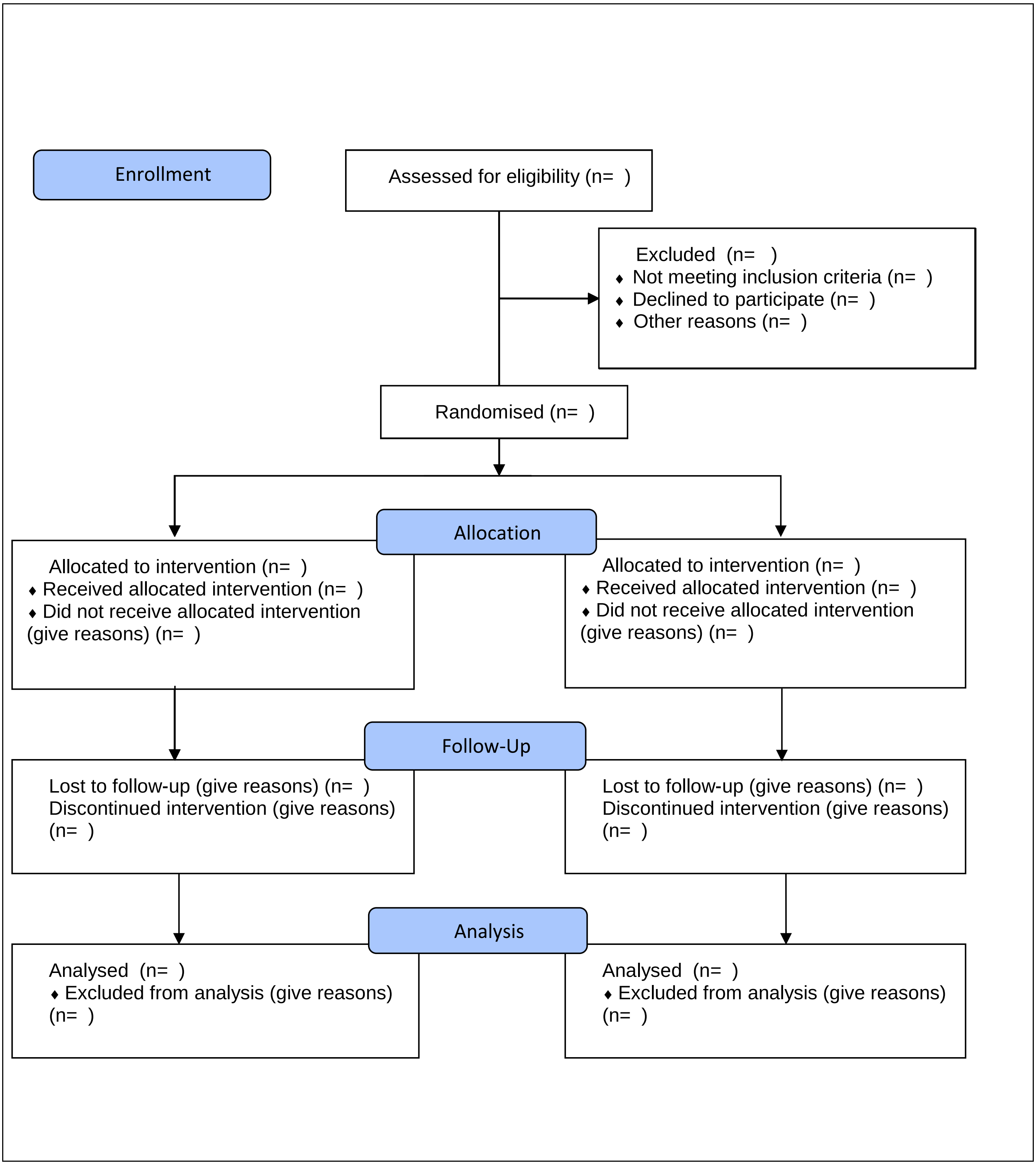

Supplement: fcz050_Supplementary_Data [file fcz050_supplementary_data.zip › Statistical_analysis_plan.pdf]
